# Invasive mosquito surveillance in the United Kingdom 2020 to 2024: First detection of *Aedes aegypti* eggs in the UK and further detection of *Aedes albopictus*

**DOI:** 10.1371/journal.pgph.0004968

**Published:** 2025-10-01

**Authors:** Colin J. Johnston, Amy C. Edwards, Alexander G. C. Vaux, Anthony J. Abbott, Harrison Hardy, Roxanna Wilson, Bathsheba L. Gardner, Arran J. Folly, Clare Strode, Daniel W. Crosgrove, Archie K. Murchie, Christopher O’Connor, Ian Rea, Kirsty Hewitt, Charlotte Flynn, Rachel Hornigold, Emma Widlake, Matthew Baylis, Marcus S.C. Blagrove, Gillian Armstrong, Jacqueline Gallavin, Annmarie Goodwin, Nicole Wakely-Griffiths, Jolyon M. Medlock

**Affiliations:** 1 Medical Entomology & Zoonoses Ecology (MEZE), Centre for Climate and Health Security, United Kingdom Health Security Agency, Porton Down, Salisbury, United Kingdom; 2 Vector-borne diseases, Animal and Plant Health Agency, Surrey, United Kingdom; 3 Department of Biology, Edge Hill University, Ormskirk, United Kingdom; 4 Sustainable Agri-Food Sciences Division, Agri-Food and Biosciences Institute, Belfast, United Kingdom; 5 South East Health Protection, United Kingdom Health Security Agency, Horsham, West Sussex, United Kingdom; 6 Electron Microscopy & Microbiology, United Kingdom Health Security Agency, Porton Down, Salisbury, United Kingdom; 7 Institute of Infection, Veterinary and Ecological Sciences, Faculty of Health and Life Sciences, University of Liverpool, Liverpool, United Kingdom; 8 Emerging Infections and Zoonoses, United Kingdom Health Security Agency, London, United Kingdom; 9 Mid Kent Environmental Health Shared Service (Maidstone Borough Council, Swale Borough Council & Tunbridge Wells Borough Council), Maidstone, United Kingdom; Mahidol University, THAILAND

## Abstract

Here, we provide an update on invasive mosquito surveillance activities coordinated by the UK Health Security Agency (UKHSA) between May 2020 and November 2024. *Aedes albopictus* and *Aedes aegypti* are invasive mosquitoes and not currently established in the UK. Both are vectors of various arboviruses including emerging pathogens such as dengue. Therefore, their introduction into the UK poses a threat to public health. In 2024, vector surveillance to detect and limit the establishment of invasive mosquito species involved 1070 ovitraps at 117 localities across England, Wales and Northern Ireland, expanding from 58 in 2019. Ovitraps at seaports, airports, and highway transport hubs were examined every two weeks, between May and October, each year. In 2020, 2021 and 2022, no *Ae. albopictus* specimens were detected. In September 2023, *Aedes aegypti* eggs were detected in a freight storage facility near Heathrow Airport. Identification of these eggs was confirmed morphologically and molecularly. Targeted enhanced surveillance yielded no further detections, suggesting an isolated incursion. In August 2024, *Ae. albopictus* was detected for the first time since 2019. Four eggs were found in an ovitrap at a service station along the M20 motorway in Kent, in South East England. In accordance with existing national contingency planning, the local authority collaborated with the landowner and UKHSA to conduct larval source reduction, and UKHSA conducted enhanced surveillance. There were no further detections of eggs or adult *Ae. albopictus* mosquitoes during the enhanced surveillance period. This project is complemented by UKHSA’s broader mosquito surveillance efforts, including deploying adult mosquito traps in 307 localities across England and Wales, and a mosquito recording scheme that accepts submissions from the public. Combined, our findings suggest that future incursions of invasive mosquitoes in the UK are likely and undertaking enhanced surveillance is key to identifying and reducing the likelihood of their establishment.

## Introduction

The proliferation and expansion of invasive mosquito species, particularly *Aedes albopictus* (Skuse, 1894) and *Aedes aegypti* (Linnaeus, 1762), in Europe poses a significant potential threat to public health due to their role as vectors for pathogens that cause diseases such as dengue fever, chikungunya, and Zika virus. Compounding this issue are the effects of climate change, which is increasing the suitable climatic range for these vectors across the European continent [1–3], including at higher latitudes. Notably, *Ae. albopictus* has made northward advances in France and is now found in the major cities of Paris, Strasbourg, and Lyon, leading to local outbreaks of autochthonous pathogen transmission, including the first recorded autochthonous dengue cases in Paris in September 2023 [4]. In 2024, France reported 82 autochthonous dengue cases between June and September, inclusive. Italy has recorded autochthonous dengue cases since 2020, with 10 cases and none in 2021 or 2022. In 2023, Italy reached a then-record high for the EU/EEA with 82 locally acquired cases. However, the 2024 outbreak in Italy’s Marche region with 199 autochthonous dengue cases [5] stands as the largest dengue outbreak ever reported in the EU/EEA and marks a significant increase in local transmission, likely driven by established *Ae. albopictus* populations, in mainland Europe [6].

The European Centre for Disease Prevention and Control (ECDC) collates data on surveillance efforts coordinated mosquito surveillance efforts across Europe to monitor the spread of invasive vector species, particularly *Ae. albopictus* and *Aedes aegypti*. Through the VectorNet project, ECDC maintains detailed distribution maps based on entomological data from national surveillance systems, scientific literature, and expert reports [7].

VectorNet data indicate that *Ae. albopictus* has expanded its range in Europe into areas with climatic conditions comparable to parts of the UK. Countries including the Netherlands, Belgium, southern Sweden, have similar isotherms to England have all reported introductions of *Aedes albopictus*, with some coastal regions of northern France seeing established populations [8].

The UKHSA (and predecessors Public Health England and Health Protection Agency) have implemented a proactive strategy, with numerous partners, to undertake active mosquito surveillance at points of entry, vehicular transport hubs and higher-risk urban centres, supplemented by a range of other activities, including citizen science led passive surveillance. The primary aim is to rapidly detect any incursion of invasive mosquito species into the UK. In addition, UKHSA provides guidance to local authority environmental health teams, empowering them to quickly implement control measures. This approach aims to prevent and delay the establishment of invasive mosquito populations, thereby effectively mitigating the risk of vector-borne pathogen transmission within the UK.

Initiated by UKHSA’s predecessor, the Health Protection Agency and Edge Hill University, invasive mosquito surveillance at UK ports began in 2010, targeting key entry points in accordance with ECDC guidance [9]. In 2016, the programme had expanded to 34 locations, where port health officers operated BG GAT and ovitraps with support from PHE and Edge Hill University. Between 2016 and 2018, inspections were carried out fortnightly from June to October, with some sites also employing BG-Sentinel or ovitrap inspections and lures [10]. In 2019, surveillance was further expanded to 56 sites—including seaports, airports, rail terminals, and roadside locations—with trapping conducted throughout the June to October period.

The surveillance first detected *Ae. albopictus* eggs in 2016 at a truck stop in Kent [9]. In subsequent years (2017–2019), *Ae. albopictus* eggs were again identified in ovitraps [11]. However, enhanced surveillance and targeted control measures prevented the establishment of larger or sustained populations, highlighting the continued importance of surveillance [10].

In parallel to the invasive mosquito surveillance, a mosquito recording scheme collects public and professional submissions of mosquito specimens and sightings, supporting early detection and helping to map the distribution of both native and non-native species, no invasive mosquitoes have been detected from this scheme [12].

This paper outlines the results of invasive mosquito surveillance conducted by UKHSA from 2020 to the end of 2024.

## Methods

The Medical Entomology and Zoonoses Ecology (MEZE) group at UKHSA conducts two annual surveillance programmes aimed at detecting incursions of invasive mosquito species. For the purposes of these programmes, an invasive mosquito is defined as any species not among the 35 currently known to be established in the UK [10]. The species most likely to be encountered include *Aedes albopictus*, *Aedes koreicus* (Edwards, 1917), and *Aedes japonicus* (Theobald, 1901). The following sections describe the two types of surveillance conducted.

### Passive surveillance for invasive mosquito: the UKHSA Mosquito Recording Scheme (MRS)

The Mosquito Recording Scheme (https://www.gov.uk/guidance/mosquitoes-how-to-report) uses passive surveillance of mosquitoes, in which members of the public submit mosquitoes (photos or specimens) for identification, year-round [12]. Although mosquito specimens and photos are submitted annually, this scheme has so far not detected any invasive mosquitoes in the UK since its inception in 2010 and received 332 records from the public between 2020 and 2024, inclusive. The surveillance is limited to volunteer contribution from the public and may miss high risk areas therefore MEZE also employs an active surveillance network using ovitraps placed at high-risk locations.

### Active surveillance for invasive mosquito using ovitraps (IMS)

**Fig 1:**
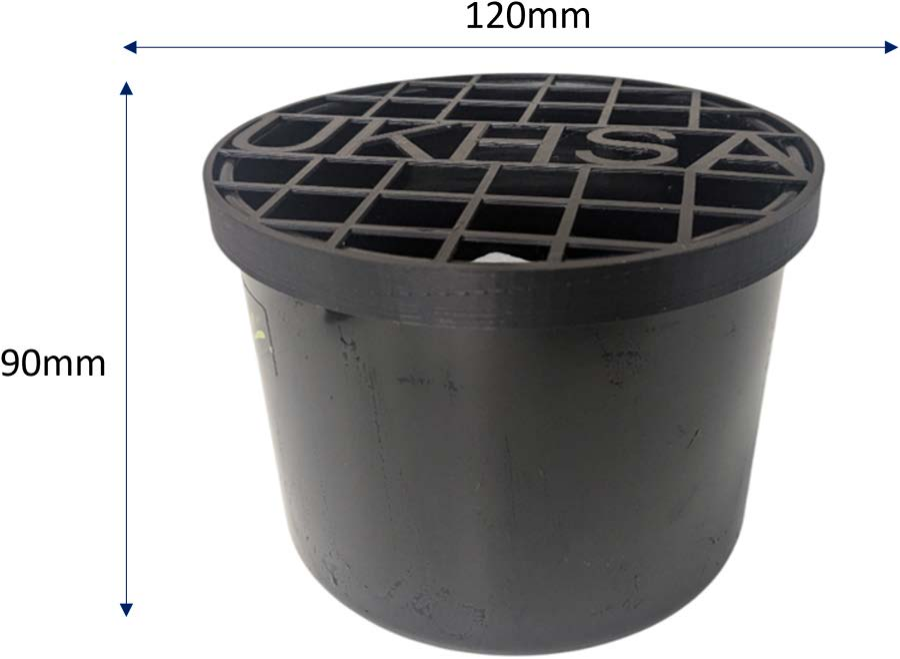
Ovitrap used for invasive mosquito surveillance with UKHSA branded 3d printed lid.

Active, trap based surveillance was employed annually during the study period, between June and October, inclusive, using the same survey methodology as in previous years [9,11]. This time period was chosen because it coincides with the peak season for invasive mosquito activity in continental Europe and with weather conditions in the UK that are conducive to mosquito development. Ovitraps consist of black plastic pots (Ramona, 120 mm radius, 90 mm in height; Luwasa, Interhydro AG, Allmendingen, Switzerland), half filled with water and containing a polystyrene block (50 × 50 × 30 mm) added as oviposition support. Where necessary, ovitraps had 3D printed lids made from black plastic (polylactide, PLA) to stop animals or the wind removing the polystyrene blocks, Fig 1. Ovitraps were placed outside with the exception of those placed in airports and warehouses that sorted imported freight. The traps were inspected at least every two weeks, while maintaining sufficiently frequent checks to prevent adult emergence in a UK climate: for reference, *Ae. albopictus*, collected from a temperate area were shown to develop from egg to adult in 36.8 ± 0.5 days at 15°C and 12.7 ± 0.2 at 25°C [13]. A full list of surveillance sites utilised between 2020 and 2024, inclusive, are found in S1 Table 1. The surveillance and response to detection was conducted by; UKHSA, local authorities, port health authorities, the Animal and Plant Health Agency (APHA) and the Agri-Food and Biosciences Institute Northern Ireland (AFBINI). Responsibilities are outlined in the UKHSA National Contingency Plan for invasive mosquitoes [14]. This provides a framework for coordinated action, should an invasive species of mosquito be identified, and were discussed in detail by Vaux *et al.,* [11].

**Fig 2:**
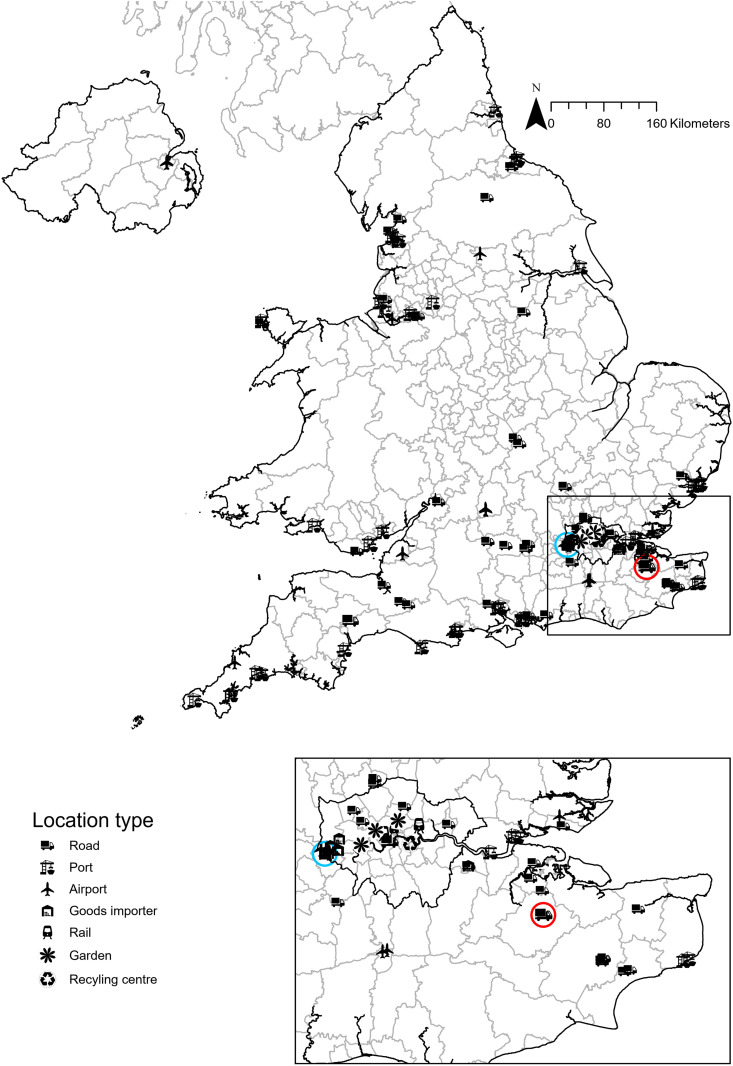
Location of targeted UKHSA invasive mosquito adult and ovitraps June-October 2024. Symbols indicate the type of site. Blue symbol shows the location of the 2023 *Aedes aegypti* detection and the red symbol 2024 *Aedes albopictus* detection. A list of locations can be found in supplementary information 1. Base map source: Local Authority Districts (May 2023) Boundaries UK BFE, Office for National Statistics licensed under the Open Government License v3.0. Contains OS data © Crown copyright and database right [2023]. Additionally, the terms of the Open Government License (OGL) are fully compatible with the CC-BY 4.0 license.

**Fig 3:**
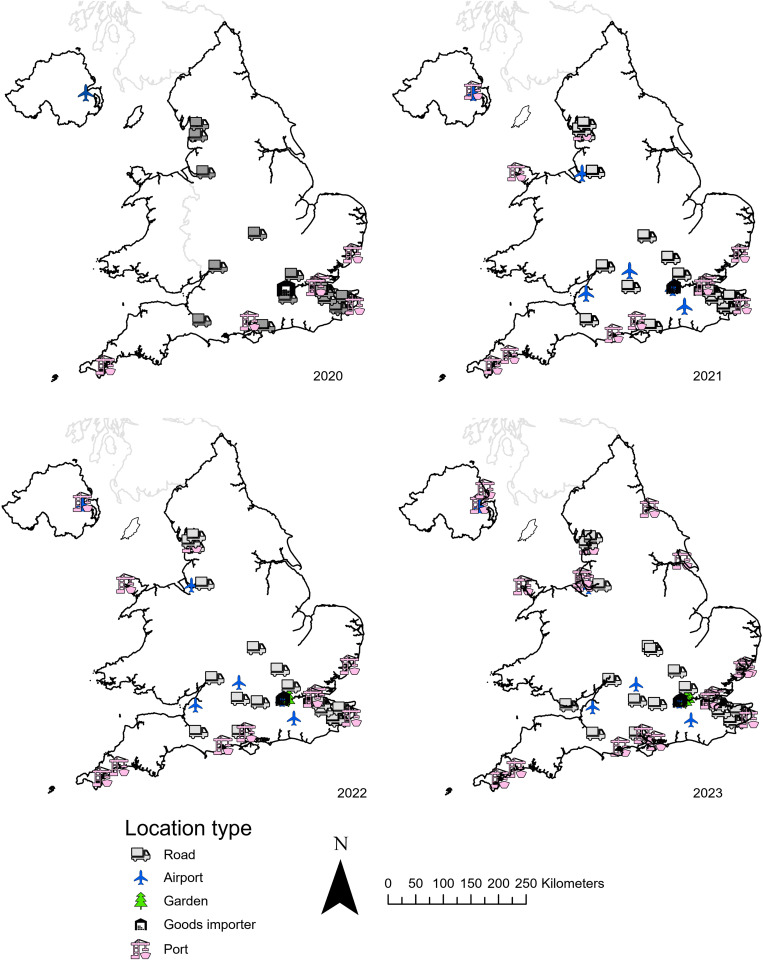
Location of UKHSA adult and ovitraps June-October 2020 to 2023, inclusive. Symbol, colour, and shape indicate the year each location had ovitraps running. A list of locations can be found in supplementary information 1.Base map source: Local Authority Districts (May 2023) Boundaries UK BFE. Office for National Statistics licensed under the Open Government License v3.0. Contains OS data © Crown copyright and database right [2023]. Additionally, the terms of the Open Government License (OGL) are fully compatible with the CC-BY 4.0 license.

Locations (Fig 2 and 3) included points of entry as suggested by the European Centre for Disease prevention and Control (ECDC) surveillance guidance [15] and outlined in Vaux & Medlock [16]. These locations were selected because they are the most likely entry points for mosquitoes into the country, and resources were available to set up and operate traps there. At each location, a minimum of five ovitraps were operated, placing the traps in locations on or adjacent to the port or airport such as along boundary fences and at freight storage facilities, baggage terminals, aircraft, gates and dockside locations; further detail on site selection and justification can be found in Vaux *et al.,* 2020 [11]. In parallel with routine surveillance, MEZE implemented additional ovitrap monitoring in designated high-risk areas to verify the absence of undetected *Aedes albopictus* populations. This targeted surveillance focused on urban green spaces—specifically parks and cemeteries—in London and along the south coast, where logistical capacity and staffing resources permitted.

### Enhanced urban park and cemetery surveillance

Climate-driven habitat suitability models suggest that, as of the 2010s, the area around London and the south coast of England is suitable for the establishment of *Ae. albopictus* populations [13]. As a result, MEZE placed additional ovitraps in potentially suitable locations, specifically urban parks and cemeteries in London and along the south coast.

**Fig 4:**
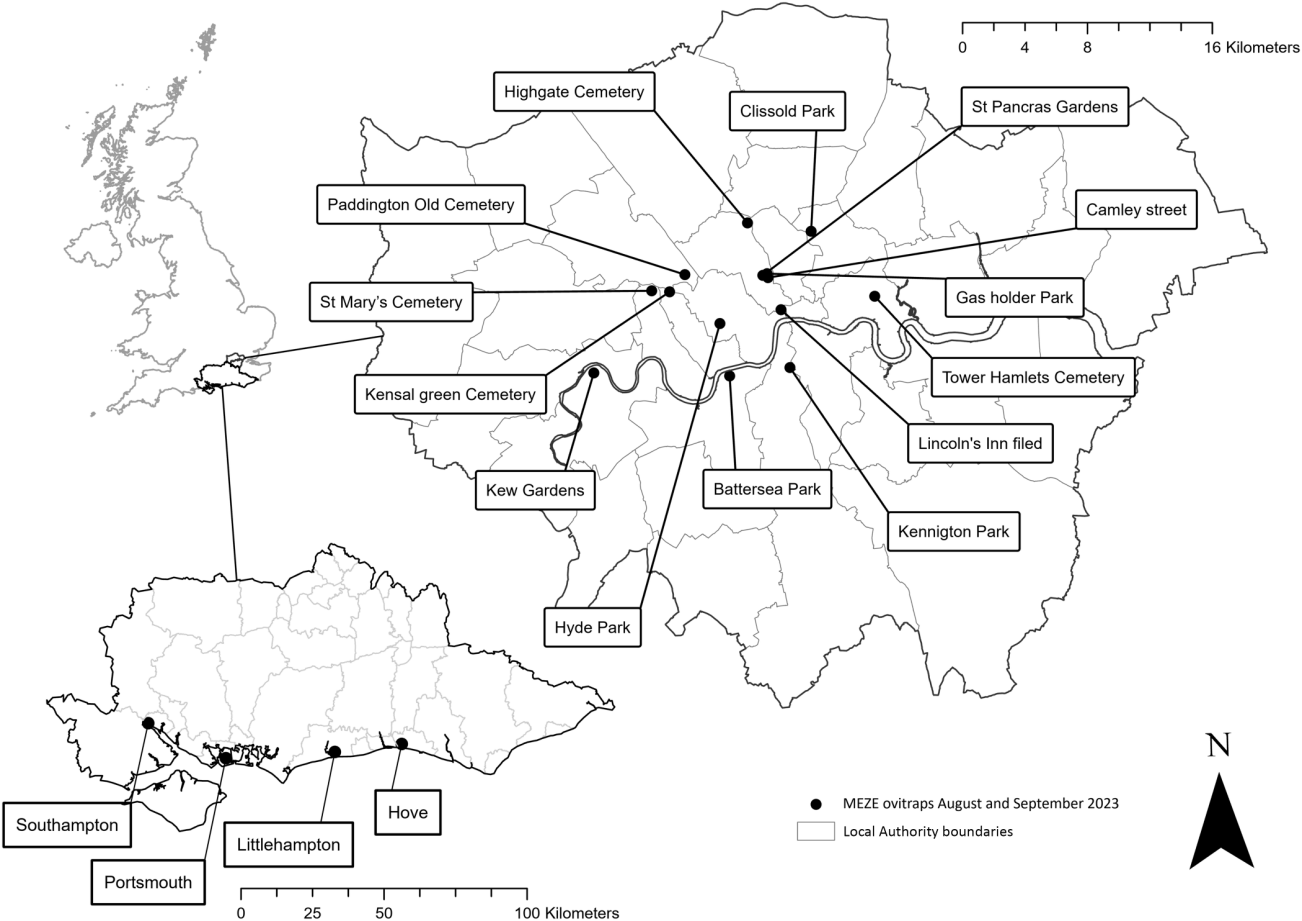
Locations of UKHSA ovitraps 2023, in the Greater London and south coast areas. Base map source: Local Authority Districts (May 2023) Boundaries UK BFE, Office for National Statistics licensed under the Open Government License v3.0. Contains OS data © Crown copyright and database right [2023]. Additionally, the terms of the Open Government License (OGL) are fully compatible with the CC-BY 4.0 license.

In August 2023, to supplement local authority (LA) surveillance, UKHSA conducted additional surveillance in these regions (Fig 4). By 2024, many more LAs across London were actively engaged in surveillance efforts, and therefore, no further supplementary surveillance was deemed necessary.

From each ovitrap, polystyrene blocks and larvae were sent to MEZE for examination. This is firstly a visual inspection with a low powered magnifying lens and then any suspected eggs are inspected using a stereomicroscope with 8–50/8–80 × magnification.

Morphological identification of material of each life stage was conducted using standard microscopy methods and established keys [17,18]. Examination of the fine structure [19] of suspect eggs was achieved using a scanning electron microscope (SEM). For SEM the mosquito egg was mounted onto an SEM stub using a sticky carbon disk. The egg was air-dried, then gold coated using an Atom Tech spurt. Suspected invasive mosquito eggs were further confirmed through molecular identification methods. The mitochondrial cytochrome oxidase subunit 1 (CO1) gene and the nuclear internal transcribed spacer 2 (ITS2) region of ribosomal DNA were sequenced from collected eggs and queried against GenBank sequences using the Basic Local Alignment Search Tool (BLAST). DNA was isolated from individual eggs, homogenized by electronic pestle, extracted using the DNeasy Blood and Tissue Kit (Qia-gen GmbH, Hilden, Germany) according to the manufacturer’s instructions and amplified by polymerase chain reaction following the methods described in Harbach *et al.,* 2017 [20]. Sequencing was carried out using an AB 3500 genetic analyser (AppliedBiosystems, Inc., Foster City, CA, U.S.A.).

### Outline of standard response to detecting eggs

Upon confirming the detection of invasive mosquito eggs, a response was enacted as outlined in the *National Contingency Plan for Invasive Mosquitoes* [14]. MEZE informed the appropriate regional UKHSA Health Protection Team (HPT), which subsequently raised a local incident. Any such meeting was attended by representatives from the HPT, Local Authority Environmental Health Officers (LA EHO), the UKHSA communications team, and MEZE. During meetings, MEZE provided an explanation of the findings and delivered technical advice on the necessary measures to minimise both the likelihood of establishment and further spread of invasive mosquitoes.

Following Incident Management Team (IMT) meetings, a site visit was conducted by MEZE and the Local Authority. Enhanced surveillance would then be implemented, including the deployment of additional traps, consisting of at least ten additional ovitraps (supplementing the existing ovitraps) and 5 BG-Sentinel traps, using Biogents (BG)-Sweetscent lure. These traps were placed at the detection site and within a 300 m radius control area. The aim of the enhanced surveillance was to determine whether the positive traps indicated the importation of a single *Ae. albopictus* female or the presence of a larger population with multiple oviposition sites, within or around the detection location. During this period, MEZE entomologists monitored all traps at four-day intervals.

Based on technical recommendations from MEZE, Local Authorities and landowners implemented appropriate control measures. These measures include the removal (and incineration) of litter, ensuring that drains were free-flowing, and, where necessary, treating drains with Aquatain (Aquatain products Pty Ltd., Australia); a silicone-based monomolecular films - combined, these measures would reduce or remove potential larval breeding sites. A Local Authority Environmental Health team led the prescribed control response over a two-week period, supported by site owners and pest contractors. MEZE maintained enhanced surveillance throughout this period to monitor progress and provide further guidance as needed.

After 14 days, with no additional detection of eggs, larvae, or adult mosquitoes, an incident is declared closed. Routine ovitrap surveillance resumes on a fortnightly basis and is continued until the conclusion of the surveillance period at the end of October.

### Additional UKHSA adult mosquito trapping projects in England and Wales

**Fig 5:**
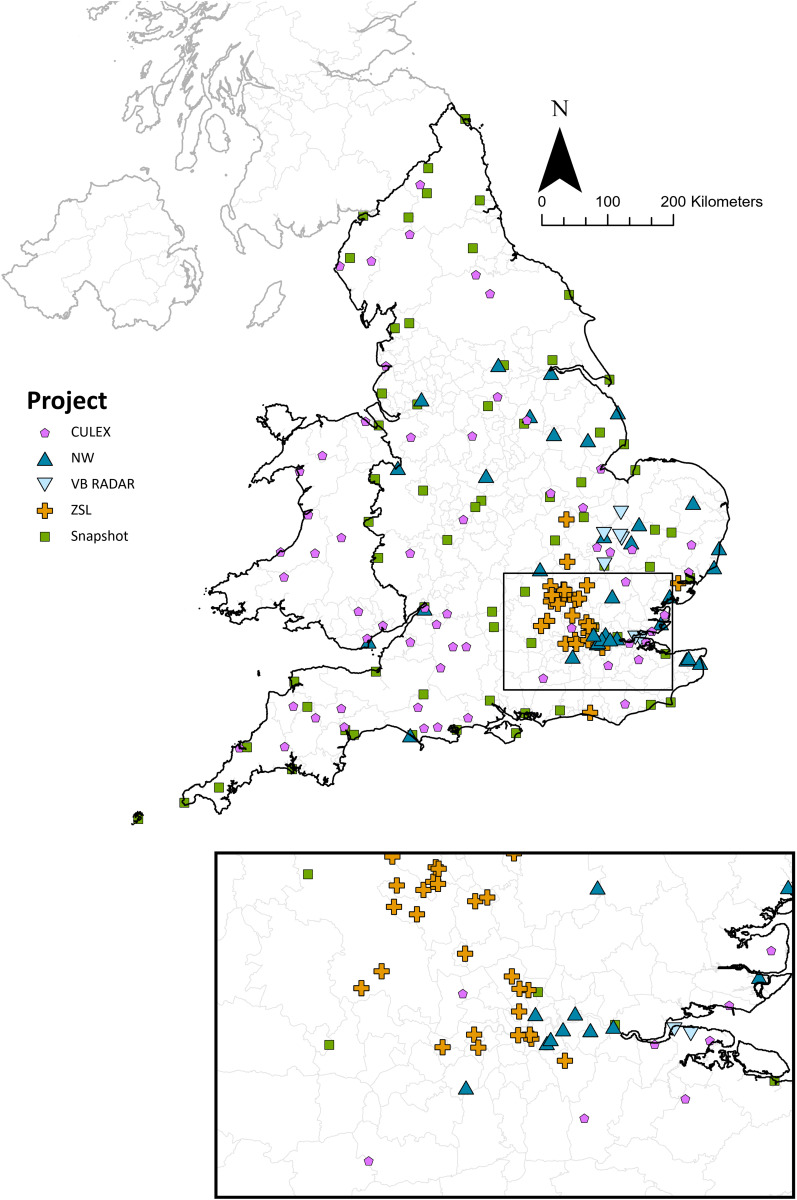
Adult mosquito traps set up by UKHSA and volunteers in 2024. Symbols represent the locations of where each trap was placed. **Base map source: Local Authority Districts (May 2023) Boundaries UK BFE,* *Office for National Statistics licensed under the Open Government License v3.0. Contains OS data © Crown copyright and database right [2023]. Additionally, the terms of the Open Government License (OGL) are fully compatible with the CC-BY 4.0 license.**

In addition to targeted invasive mosquito surveillance, MEZE also conducted other adult mosquito surveillance projects using methodology suitable for detecting IMS. During 2024, these additional activities equate to trapping for adult mosquitoes at 307 localities (Fig 5). Although these projects did not specifically target invasive mosquitoes, the traps used have been shown to be effective at collecting *Ae. albopictus*. Full data and methods will be published elsewhere. The following is a summary of the trapping methods.

### Nationwide mosquito project

**Fig 6:**
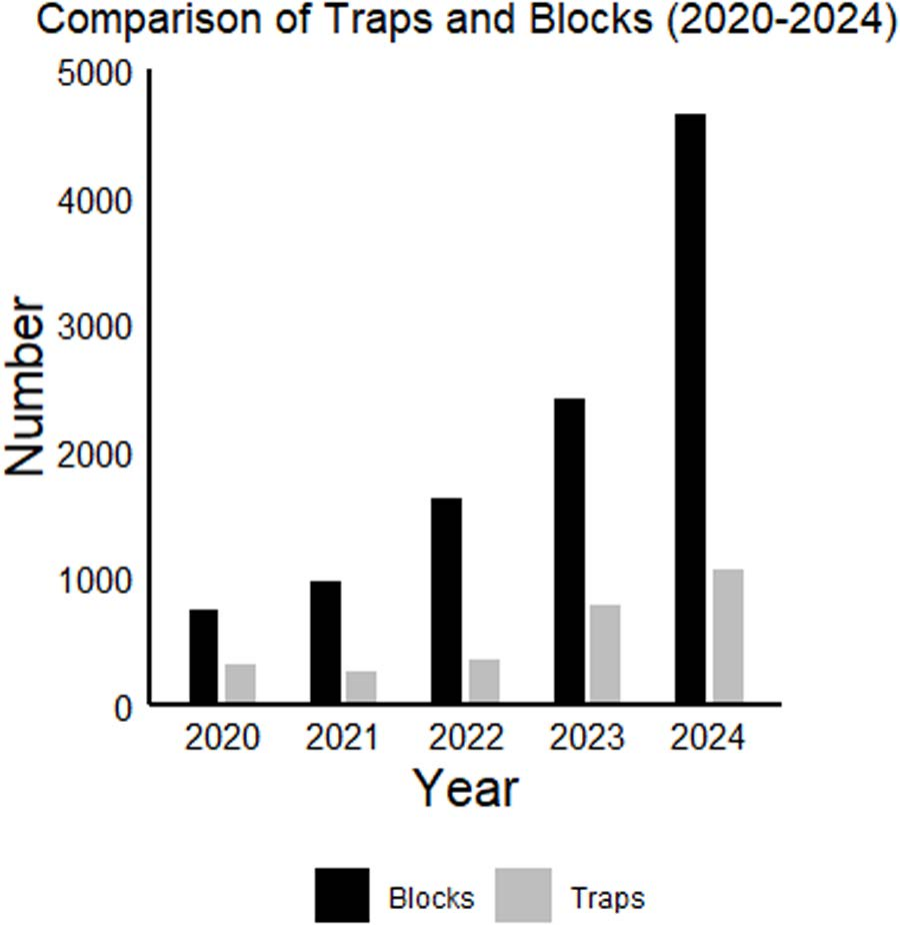
Number of ovitraps and numbers of blocks for UKHSA invasive mosquito surveillance for 2020 to 2024.

Annual adult mosquito surveillance across England, referred to as Nationwide used Mosquito Magnet traps (Mosquito Magnet® Executive model, Woodstream Corporation, St. Joseph, MO, USA) baited with an octenol (1-Octen-3-ol) lure and was run at 37 wetland sites (Fig 6) in 2024 (12 in 2020, 14 in 2021, 11 in 2022, and 13 in 2023).

### CULEX project

In 2023, adult mosquito traps were operated across 200 sites across England and Wales, covering a range of land-use types, as part of a UKRI-Defra funded project led by the University of with MEZE. A sentinel-style BG-Pro trap (Biogents AG, Regensburg, Germany) baited with BG-Lure and BG-CO_2_ Generator (Biogents AG, a yeast-derived CO_2)_*,* and a gravid *Aedes* trap (Biogents AG, Regensburg, Germany) utilising a sticky card and hay-infused water, were used. One of each trap type was placed in each sampling location and collected after three nights and removed. In 2024, BG-Pro traps were deployed at 60 sites and in addition, larval sampling was performed at each site. All mosquitoes captured were identified by staff at MEZE and the University of Liverpool using standard morphological keys [21].

### Snapshot mosquito survey

Volunteers from 72 sites across England were enlisted to operate a BG-Pro mosquito trap, and BG-CO_2_ Generator, in each 50km grid-square for two separate three-night trapping sessions: the first in late August and the second in early September 2024. All mosquitoes captured were identified by MEZE staff using standard morphological keys.

### Vector-Borne real-time arbovirus detection and response (VB-RADAR) project

This UKRI-Defra funded project led by the Animal Plant Health Agency is focusing on the surveillance of Orthoflaviviruses in mosquitoes and birds. Mosquito sampling was conducted at 16 wetland sites with MEZE around Kent in 2023 (Dorset, Kent, Essex, Suffolk, London), and at five wetland nature reserves in Cambridgeshire and two sites in the Kent marshes in 2024. BG-Sentinel traps baited with BG-Lure and BG-CO_2_ Generator were deployed at all sites, with Mosquito Magnets additionally used at two Kent locations sampled in 2023 and 2024. In 2024, 33 staff working for the Zoological Society of London (ZSL) were recruited to run a Biogents BG-Pro baited with BG-Lure and BG-CO_2_ Generator in their gardens once a week, every other week from July to October.

## Results

Between 2020 and 2024 surveillance effort significantly increased with the number of polystyrene blocks returned from ovitraps increasing each year (Fig 6), with slight variations in the start date due to limitations in staff resources.

### Active invasive mosquito ovitrap surveillance

#### 2020.

In 2020, surveillance was conducted between weeks 21 and 41, presumed to be peak mosquito activity in UK, at 25 locations (16 vehicular transport hubs, and nine seaports), and monitored by 20 local authorities. At the conclusion of the surveillance period, a total of 250 ovitraps and 750 blocks had been checked. No detections of eggs, larvae, or adults of any invasive mosquitoes (*Ae. albopictus, Ae. aegypti, Aedes japonicus* and *Aedes koreicus*). *Culex pipiens* s.l. Linnaeus, a common British species was found on six occasions. This year marked a significant reduction in surveillance compared to 2019 (as described in Vaux *et al.,* 2020, [11]) due to social-distancing restrictions imposed in the UK during the COVID-19 global pandemic.

#### 2021.

Surveillance occurred between weeks 21 and 42. A total of 265 mosquito ovitraps were set up across 31 locations (two airports, four importers, 21 vehicular transport hubs, and four seaports), and monitored by 16 local authorities. By the end of the surveillance period 959 blocks had been checked. There were no detections of eggs, larvae, or adults of any invasive mosquitoes. The native British species *Culex pipiens* s.l. larvae were found on eight occasions. As with 2020 this year saw a reduction in surveillance compared to 2019, due to the ongoing impact of the COVID-19 pandemic.

#### 2022.

In 2022 surveillance occurred between weeks 23 and 41: 28 organisations deployed ovitraps at 44 sites (seven airports, 11 seaports, and 26 vehicular transport hubs). A total of 350 ovitraps were set up and 1622 polystyrene blocks were checked. No *Ae. albopictus* or invasive mosquito species eggs, larvae or adults were found at any location.

#### 2023.

Surveillance was conducted by 31 organisations at 57 sites (22 seaports, seven airports, and 28 vehicular transport hubs) by UKHSA partners between weeks 23–43. Additionally, ovitraps were also deployed at four cemeteries along the South Coast of England (Fig 3) and 14 parks and cemeteries in Central London by MEZE (Fig 4) in August and September. A total of 775 ovitraps were set up and 2411 blocks were checked. On 14^th^ September, at Tower Hamlets Cemetery, London, 83 eggs were found on an oviposition substrate block but these were suspected, based on morphology and subsequently confirmed molecularly, as *Aedes geniculatus* (Olivier, 1791); a British native dendromlimnic (tree-hole) species and no further action was taken. No *Ae. albopictus* eggs, larvae, or adults were found at any other location. Two *Ae. aegypti* eggs were found in a single ovitrap at a location near London Heathrow Airport (Fig 2). No other invasive mosquito species eggs, larvae or adults were found at any location.

### 2023 *Aedes aegypti* detection at London Heathrow Airport

On 6^th^ September, two eggs were found in an ovitrap located about 30 metres from the external doors of a freight storage facility near London Heathrow Airport. This facility handled airfreight, with cargo potentially originating from Asia or Africa and transiting through Istanbul. In response, the plans outlined by the *National Contingency Plan for Invasive Mosquitoes* (described above) were enacted. Additional traps were strategically placed in the centre, along the edges, and immediately outside the building in which the suspected invasive mosquito eggs were found. The site appeared relatively clean (and inimical for aquatic mosquito development), with very few suitable habitats both inside and in the adjacent area. A bucket containing water, was identified and removed, and some litter in the parking area was noticed, prompting local authorities to liaise with the owner to address these issues. The IMT focused mosquito surveillance efforts on the site and its surrounding parking area. The eggs were initially suspected as *Ae. aegypti* (based on origin of cargo), and later confirmed by DNA barcoding. Within 24 hours, enhanced surveillance was deployed and continued for two weeks, and no adults or additional eggs were detected, suggesting an isolated incursion. Egg rafts, larvae, and *Culex pipiens* s.l. adults were found in a water-filled bucket, which was then emptied and turned upside down.

#### 2024.

Surveillance was conducted by 70 organisations between weeks 23–44 at 117 sites which consisted of 22 seaports, six airports (including all four Heathrow terminals, seven importers, and two inspection posts in and around London Heathrow Airport), 68 vehicular transport hubs, two gardens, and four railway stations. A total of 1070 ovitraps were deployed and 4650 blocks were checked. *Ae. albopictus* eggs were found at one site in Kent, Southeast England (Fig 2).

### 2024 Aedes albopictus detection in Maidstone

On 19^th^ August, a positive polystyrene block was identified from an ovitrap placed at a motorway service station along the M20 in Kent, where four eggs were detected. The trap was located within ten metres of a fast-food drive-through order point and hidden in undergrowth. The service station, situated 40 miles northwest of the international port of Dover, is privately owned and includes a lorry park catering to cross-channel freight, a hotel, fuel station, car park, lorry park, and restaurants used by members of the public.

The day after the detection, MEZE attended the site with the local authority, and a multi-agency response was led by the UKHSA South East Health Protection Team (SE HPT), and an Incident Management Team (IMT) was formed. Attendance included members from SE HPT, MEZE, UKHSA Emerging Infections and Zoonoses, Emergency Preparedness, Resilience, and Response (EPRR), and Communications teams from UKHSA, along with Environmental Health, Public Health, and Communications teams from the Mid-Kent Environmental Health Shared Service on behalf of Maidstone Borough Council.

Litter clearance (and subsequent incineration) at the site was facilitated by the Mid-Kent Environmental Health Shared Service, and it was carried out by both the local authority and motorway site facilities teams. A drain survey was conducted, and although no larvae were found, the drains were treated with a polydimethylsiloxane product (Vazor Provecta, distributed by Killgerm Chemicals Ltd and manufactured by KB Pharma in Abano Terme, Italy). Enhanced mosquito surveillance was carried out by UKHSA, with 15 additional ovitraps and three adult BG-Sentinel traps installed around the site, operated continuously for the next two weeks. In accordance with the national contingency plan all existing and additional traps were checked every three days, After 14 days, with no further eggs, larvae, or adults detected, the incident was closed, and routine ovitrap surveillance resumed fortnightly until the end of October. There was no further evidence of *Ae. albopictus,* which were confirmed by DNA barcoding.

### Adult trapping projects and citizen science

The 100 gravid aedes traps (GATs) and 100 BG Pro traps set up in 2023 and the 307 adult traps operated in 2024 as part of the VB-RADAR, Nationwide, Culex and ZSL projects did not detect the presence of *Ae. albopictus* or any other invasive mosquitoes throughout the sampling period. The citizen science led MRS did not receive any submissions of invasive mosquito samples and only recorded British species. Other species that were recorded for these projects are reported elsewhere (up to end of 2021) published by Johnston *et al*. [12].

## Discussion

Over the past five years (between May 2020 and November 2024), UKHSA has significantly enhanced its long-standing invasive mosquito surveillance (since 2010) in response to the rising risk of incursion, posed by *Ae. albopictus* and other mosquito species that can transmit animal and human pathogens.

With increasing populations of these mosquitoes recorded across Europe, the primary objective of the surveillance is to detect early incursions and prevent the establishment of invasive vector species within the UK, to mitigate and reduce the likelihood of autochthonous transmission of arboviruses such as dengue, chikungunya and Zika which occurred in France, Italy and Spain.

Before 2020, invasive mosquito species had been detected for four consecutive years (2016–2019), on six occasions, and this trend was anticipated to continue. However, the COVID-19 pandemic resulted in a significant reduction in human travel and imports into the UK [22] and between 2020 and 2022 no invasive mosquitoes were found at any surveillance locations. In 2023, the surveillance expanded to 24 extra localities and found no evidence of invasive mosquitoes.

The COVID-19 pandemic-related travel restrictions and social distancing policies likely reduced the frequency of mosquito incursions into the UK by limiting the volume of international passenger and cargo movement, key pathways for the unintentional introduction of invasive species. Reduced human mobility, especially air and vehicle traffic from endemic regions, meant fewer opportunities for mosquitoes such as *Ae. albopictus* to hitchhike into new areas. Additionally, operational limitations at ports and transport hubs may have altered mosquito surveillance intensity and coverage. Studies have similarly reported a temporary decline in the spread of invasive vectors during COVID-19 lockdowns, attributing this trend to reduced travel and cross-border movement [23].

By 2024, the surveillance network had expanded considerably, with the number of participating sites increasing from 24 in 2021–24 in 2022, 58 in 2023, and 117 localities in 2024. Despite these extensive trapping efforts at locations deemed at high risk of incursion, across England, Wales, and Northern Ireland; a UK-wide Mosquito Recording Scheme; and the deployment of over 300 adult mosquito traps—only two detections of invasive mosquito species were reported during this period.

The detection of two *Ae. aegypti* eggs in an ovitrap at a freight storage facility near London Heathrow Airport is a noteworthy finding. The only previous known incursions of this species are: an adult male discovered in near Liverpool in 2017 [24]; a suspected 7–10 mosquitoes linked to sailors infected with Yellow Fever in Swansea port, Wales, in 1865 [25]; and curiously larvae found in a beech tree rot hole in Epping Forest in 1919 [26]. The UK’s climate, particularly its cold winters, limits the survival of adult *Ae. aegypti* and the overwintering of their eggs. Without prolonged warm temperatures, the lifecycle of *Ae. aegypti* cannot be sustained. At temperatures below 14–15°C, *Ae. aegypti* becomes less active and struggles to feed on blood, as shown in several studies [27,28]. Adult *Ae. aegypti* typically cannot survive more than 2–3 days without a blood meal in warmer tropical conditions, longer periods of cold weather below 14–15°C are likely to cause death before the return of more favourable conditions [29]. Consequently it is commonly found only in tropical and subtropical regions around the world, mainly between latitudes 35°N and 35°S, these regions roughly correspond to a winter isotherm of 10°C [30]. Unlike *Ae. albopictus*, which can lay cold-resistant eggs capable of entering diapause to endure unfavorable environmental conditions, *Ae. aegypti* eggs lack this physiological adaptation. Studies indicate that *Ae. aegypti* eggs are highly sensitive to prolonged cold temperatures, with temperatures below 10°C significantly reducing their viability and freezing temperatures typically proving lethal [27,29]. Therefore, the establishment of self-sustaining populations of this species in the UK seems unlikely under present climatic conditions [24,31].

In 2024, the detection of *Ae. albopictus* marked the seventh instance of this species being identified in the UK since 2016. The positive ovitrap was located near a fast-food drive-through at a motorway service station. Given that *Ae. albopictus* has been documented traveling inside vehicles [32], it is hypothesized that *Ae. albopictus* may have arrived via a vehicle from mainland Europe and exited when the vehicle stopped. These high-traffic fast-food locations had not previously been prioritized for targeted surveillance, highlighting the importance of monitoring such areas where invasive mosquitoes could inadvertently be introduced. Critically, no additional eggs, larvae, or adults were found in other traps during routine and enhanced surveillance, nor in any discarded water-filled containers, suggesting that this was a transient incursion from outside the UK.

Additionally, traps operated by UKHSA in 2023 in London and along the Southeast coast were strategically placed in areas with suitable habitats to provide evidence that *Ae. albopictus* was not already present in climate permissible urban areas; none of these traps detected invasive mosquitoes. The increased number of LAs operating ovitraps in the London area, on behalf of the UKHSA, meant the 2023 UHKSA-led survey was not repeated in 2024. This further indicates that there is currently no evidence that *Ae. albopictus* is widely established in central London or along the South Coast of England or that it forms transient localised populations, corroborated by the absence of reports for invasive mosquitoes sent to the UK Mosquito Recording Scheme [12].The Mosquito Alert system [33] has mapped the European spread of *Ae. albopictus*, uncovering evidence of its vehicle-facilitated dispersal [32], and documenting the first detection of *Ae. japonicus* in the region [28]. This platform has since been expanded to include multiple European nations, including the UK, offering an additional avenue for public participation and submissions, this system has 204 records [34] of UK mosquitoes and has not to date, detected *Ae. albopictus*. The citizen science project iNaturalist, coordinated by UKHSA, provides data about mosquitoes in the UK [35] and has 1629 records of mosquitoes in the UK [36] none of which have been confirmed as an invasive mosquito. Consequently, there is no evidence to date to suggest that any invasive mosquito species have established in the UK.

For UK, climate modelling has highlighted the increasing climate suitability of habitats for invasive mosquito species like *Ae. albopictus* across southeast England [1,2,37]. Rising temperatures, milder winters, and extended growing seasons have facilitated the northward expansion of these vectors, previously restricted to warmer climates in mainland Europe [2].

Habitat suitability models predict that, *Ae. albopictus* could establish populations in southern England in the coming decades due to warming climates and increased precipitation, which provide more breeding sites [38]. These species distribution models forecast potential range expansions [39], underscoring the need for increased surveillance.

The changing distribution of mosquito vectors has important implications for animal and public health in the UK. As urban areas become more climatically favorable for species such as *Ae. albopictus*, the risk of arboviral diseases like dengue, Zika, and chikungunya increases [40]. Although these diseases are not currently endemic in the UK, 634 travel-associated dengue cases were reported in 2023 [41] and 473 cases were recorded in the first half of 2024 [42]. Sporadic outbreaks could occur due to the importation of infected individuals and the presence of competent mosquito vectors [38] as has been observed regularly in other European countries. Moreover, warmer temperatures can accelerate the replication rates of arboviruses within mosquito vectors, potentially shortening extrinsic incubation periods and enhancing transmission efficiency [43]. For instance, arboviruses such as dengue and chikungunya may become more readily transmitted during prolonged warm seasons [44]. Increased precipitation and flooding events associated with climate change also provide additional novel breeding environments for mosquitoes, further facilitating their proliferation and the potential spread of associated pathogens [45].

The UK has already recorded isolated detections of *Ae. albopictus* at transport hubs, underscoring the role human activity plays in facilitating arthropod vector introductions [9]. This trend highlights the importance of proactive measures, such as enhanced surveillance at ports of entry and public awareness campaigns to mitigate risks associated with invasive mosquito species.

Both *Ae. aegypti* and *Ae. albopictus* are significant vectors globally and the economic cost of Aedes-borne diseases is rising, with cost of damage being ten times higher than that the cost of management [46], highlighting the importance of and requirement for MEZEs ongoing surveillance programme in the UK. This initiative complements other mosquito monitoring efforts, including citizen science projects and MEZE adult mosquito projects across England. So far, no invasive mosquitoes have been detected beyond recent cases, reported here, suggesting a low public health risk. However, these repeat detections underscore the need for sustained investment in surveillance and control. Strengthening monitoring networks, developing climate-resilient vector management strategies, and deploying innovative tools are crucial to preventing establishment of exotic arthropod vectors. Early intervention through surveillance is cost-effective [47] reducing the likelihood of long-term control measures and mitigating risks associated with arthropod-borne diseases like dengue and chikungunya. Without timely action, the UK faces the risk of invasive mosquito populations becoming established. As we have demonstrated here, proactive measures enhance resilience against emerging vector-borne disease risks.

## Supporting information

S1 Table Invasive mosquito surveillance locations. (XLSX)
