# Correction: Invasive mosquito surveillance in the United Kingdom 2020 to 2024: First detection of *Aedes aegypti* eggs in the UK and further detection of *Aedes albopictus*

**DOI:** 10.1371/journal.pgph.0005732

**Published:** 2025-12-30

**Authors:** Colin J. Johnston, Amy C. Edwards, Alexander G. C. Vaux, Anthony J. Abbott, Harrison Hardy, Roksana Wilson, Bathsheba L. Gardner, Arran J. Folly, Clare Strode, Daniel W. Cosgrove, Archie K. Murchie, Christopher O’Connor, Ian Rea, Kirsty Hewitt, Charlotte Flynn, Rachel Hornigold, Emma Widlake, Matthew Baylis, Marcus S. C. Blagrove, Gillian Armstrong, Jacqueline Gallavin, Annmarie Goodwin, Nicole Wakely-Griffiths, Jolyon M. Medlock

The sixth author’s name is spelled incorrectly. The correct name is: Roksana Wilson. The correct citation is: Johnston CJ, Edwards AC, Vaux AGC, Abbott AJ, Hardy H, Wilson R, et al. (2025) Invasive mosquito surveillance in the United Kingdom 2020 to 2024: First detection of *Aedes aegypti* eggs in the UK and further detection of *Aedes albopictus*. PLOS Glob Public Health 5(10): e0004968. https://doi.org/10.1371/journal.pgph.0004968.

The tenth author’s name is spelled incorrectly. The correct name is: Daniel W. Cosgrove.
